# An Automated, Gravity-driven CSF Drainage System Decreases Complications and Lowers Costs

**DOI:** 10.7759/cureus.1009

**Published:** 2017-02-03

**Authors:** Robert E Lieberson, Jan Eckermann, William Meyer, Tung Trang

**Affiliations:** 1 Department of Neurosurgery and Kern NeuroScience Institute, Kern Medical Center; 2 Department of Otolaryngology, Kern Medical Center

**Keywords:** base of skull surgery, cerebrospinal fluid, csf, complications, drainage/methods, dural tear, external lumbar drain, lumbar drain, reconstructive surgical procedures/methods, thoracic aorta surgery

## Abstract

Background: FlowSafe^TM^ (BeckerSmith Medical, Irvine, CA, USA) is a novel, robotic, external lumbar drainage (ELD) system, which was designed to control cerebrospinal fluid (CSF) drainage, reduce complications, and decrease treatment costs.

Methods: Forty-seven consecutive neurosurgical patients requiring ELD were treated using the FlowSafe system.

Results: In 39 of 40 patients with traumatic and surgical dural openings, potential CSF leaks were avoided. In seven patients with suspected normal pressure hydrocephalus, post-infectious ventriculomegaly, or pseudotumor cerebrum, we were able to assess the likelihood of improvement with shunting. The system, therefore, produced what we considered to be the “desired result” in 46 of 47 patients (98%). Our one treatment failure (2%) involved a patient with unrecognized hydrocephalus who, following a Chiari repair with a dural patch graft, was drained for six days. A persistent CSF leak eventually required a reoperation. Two patients (4%) described low-pressure headaches during treatment. Both responded to temporarily suspending or reducing the drainage rate. We saw no complications. Required nursing interventions were minimal.

Conclusions: The FlowSafe system was safe and effective. In our experience, there were fewer complications compared to currently available ELD systems. The FlowSafe was well tolerated by our patients. The near elimination of nursing interventions should allow lumbar drainage to be delivered in less costly, non-intensive care unit settings. Larger trials will be needed.

## Introduction

Commercially available external lumbar drainage (ELD) systems are primitive and essentially identical to the pressure-regulated drains first described in 1963 [1]. We describe a novel robotic system that can lower complications and costs. ELD is used to treat spontaneous and iatrogenic dural openings [2-3], to determine if shunting might be of benefit in patients with ventriculomegaly or pseudotumor [4-6], for patients following subarachnoid hemorrhage [7-9], and to decrease intracranial pressure [10]. It is used by vascular surgeons to lower the risk of spinal cord injury during aortic repair [11-12]. Reported complication rates are high and include over- and under-drainage, mechanical failure, and infection [2-3, 9, 13]. Furthermore, the management of currently available systems is costly, requires intensive care unit (ICU) or step-down level care, and involves frequent nursing interventions. An early electronic ELD system offered improved safety, but its widespread use was limited by technical issues [2, 14]. A more recently described, inexpensive, flow-regulated ELD system claimed a similar safety profile but was difficult to assemble and was not suitable for patients with increased intracranial pressure [15]. The system we trialed was easily connected and fully programmable, designed to prevent over- and under-drainage, allowed patients to remain ambulatory during treatment, required almost no oversight, and could have been used outside of the ICU setting.

## Materials and methods

This study was approved by the Kern Medical Institutional Review Board (approval #11013). Informed consent was obtained from each of 47 consecutive patients requiring ELD who presented between 2012 and 2015. Diagnoses included traumatic, spontaneous, planned or unintentional dural openings, normal pressure or post-infectious hydrocephalus, or pseudotumor.

**Figure 1:**
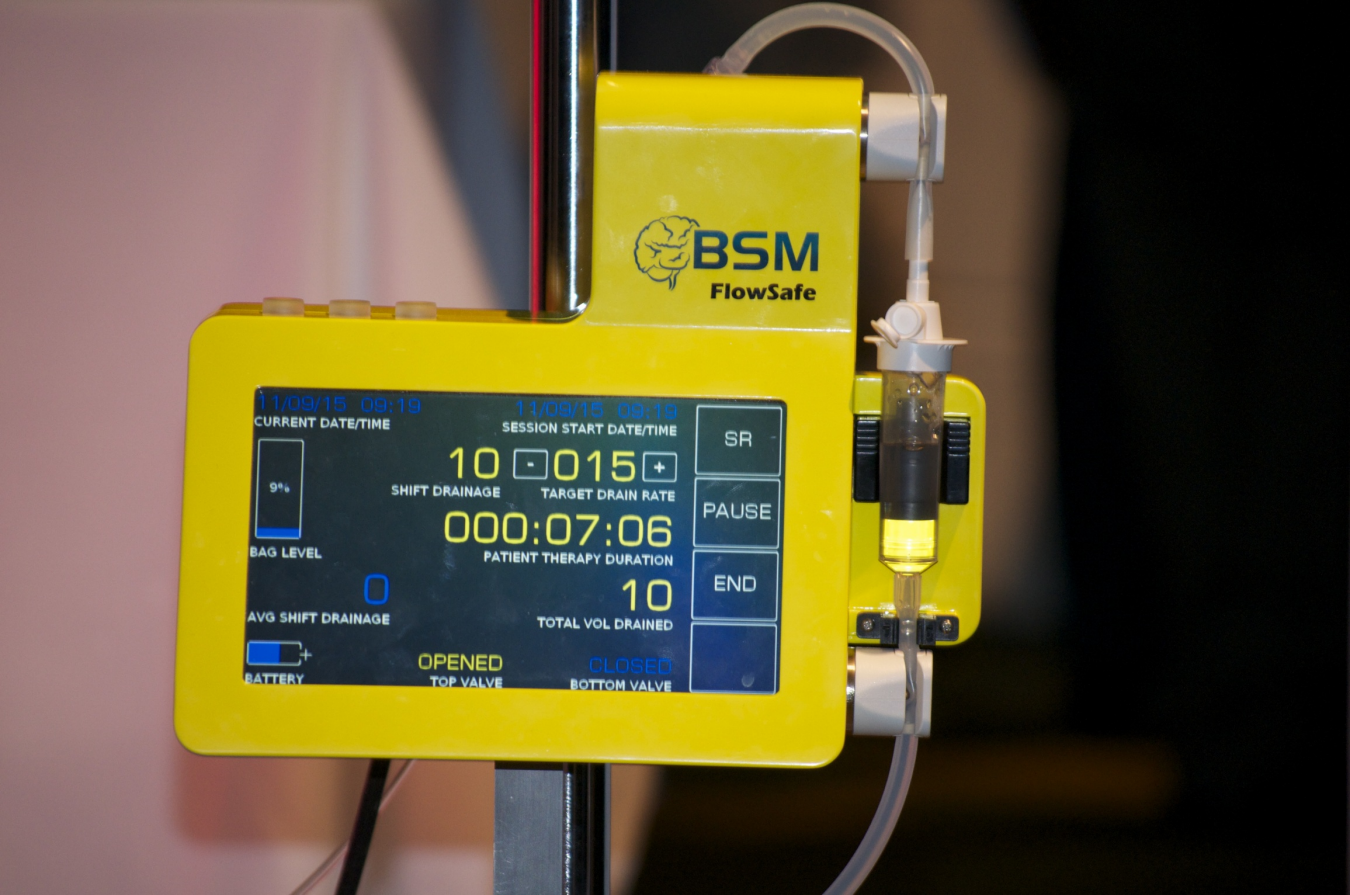
Picture of the automated lumbar drainage system

The FlowSafe^TM^ lumbar drainage system (for investigational use only) (BeckerSmith Medical, Irvine, CA, USA) is automated and gravity-driven. It consists of a compact robotic module (processor/display/flow control) and proprietary tubing (Figure 1). The robotic module includes a microprocessor, sensors, and two solenoid-controlled valves, one above and one below a calibrated drip chamber. A rate of 10 ml/hour is typical, but settings can range from 5 to 25 ml/hour. The robotic module and proprietary tubing were connected to a standard lumbar drainage catheter in our patients. Lumbar catheters (Integra Life Sciences, Plainsboro, NJ, USA) were inserted at L3-L4, L4-L5, or L5-S1 using a sterile technique and a 13-gauge Touhy needle. Approximately 15 cm of tubing was advanced into the subarachnoid space. The catheters were secured with Tegaderm dressings (3M, Saint Paul, MN, USA) before they were connected to the system’s proprietary tubing. The robotic module was placed below the level of the catheter’s insertion point. In this study, a pre-production prototype was used for the first 45 patients and a production unit was used for the remaining two. The head of the bed was positioned for comfort. Patients did not need to remain at bed rest, could position the head of the bed ad lib, and were allowed restroom privileges.

All patients were monitored for complications with serial examinations. CSF was sent for cell counts, chemistry, and culture at placement, every three days, and if an infection was suspected. Demographics, diagnoses, specifics regarding the drainage, and complications are listed in Table 1. The “desired effect” was defined as either the absence of CSF leakage or a determination that shunting might or might not be helpful in patients with suspected normal pressure, post-infectious hydrocephalus or pseudotumor, not disproven by subsequent surgical or conservative treatment.

**Table 1:** Patient Demographics and Diagnosis as well as CSF Drain Rate and Length CP - cerebropontine; CSF - cerebrospinal fluid: TB - tubercular

	Age	Sex	Diagnosis	Rate	Hrs.	Comments
1	30	F	Acoustic Neuroma Resection	10	48	
2	29	F	CP Angle Epidermoid Resection	10	71	
3	36	M	Gunshot C7-T1, CSF Leak	5	140	
4	30	F	Dural Tear, Lumbar Laminectomy	10	140	
5	58	F	Spontaneous Rhinorrhea	10	84	
6	34	M	TB Meningitis, Papilledema	5	144	
7	45	F	Pituitary Adenoma	10	50	
8	20	F	Pituitary Adenoma	10	48	
9	49	F	Dural Tear, Lumbar Laminectomy	10	120	Headache, resolved with decreased rate.
10	51	M	Dural Tear, Lumbar Fusion	10	48	
11	57	M	Pituitary Adenoma	5	48	
12	37	F	Transoral Odontoidectomy	10	48	
13	29	M	Chiari I Repair	10	144	Headache, resolved with decreased rate.
14	48	F	Dural Tear, Lumbar Laminectomy	10	48	
15	67	M	Craniofacial Trauma	10	144	
16	27	M	Craniofacial Trauma	5	144	
17	59	F	Dural Tear, Lumbar Fusion	5	120	
18	55	M	Clivus Meningioma	5	24	
19	44	F	Dural Tear, Lumbar Laminectomy	5	144	
20	20	M	Gunshot Wound to Skull Base	5	144	
21	29	M	Gunshot Wounds x5 to Skull Base	5	96	
22	66	M	Normal Pressure Hydrocephalus	5	72	
23	57	M	CP Angle Meningioma Resection	5	120	
24	22	M	Juvenile Pilocytic Astrocytoma	15	120	
25	63	M	Pituitary Adenoma	5	96	
26	43	F	Pituitary Adenoma	10	48	
27	37	M	Subdural, Postop CSF Leak	10	48	
28	52	F	Epidermoid, Endonasal Approach	10	96	
29	59	F	Pituitary Adenoma	10	96	
30	52	F	Pituitary Adenoma	10	96	
31	32	M	Pituitary Adenoma	5	72	
32	51	F	Normal Pressure Hydrocephalus	10	72	
33	32	M	Pituitary Adenoma	5	72	
34	38	M	Pituitary Adenoma	10	96	
45	33	M	Craniofacial Trauma	5	168	
36	53	F	Pituitary Adenoma	10	96	
37	40	F	Pituitary Adenoma	10	96	
38	34	M	Chiari I Repair	10	144	Persistent leak, required re-op, shunt.
39	23	M	Craniofacial Trauma	10	144	
40	26	F	Pseudotumor Cerebrii	10	72	
41	60	M	Cocci Meningitis, Hydrocephalus	10	48	
42	48	M	Normal Pressure Hydrocephalus	10	48	
43	34	M	Dural Tear, Lumbar Laminectomy	10	168	Prototype device required service.
44	46	F	Cerebellar Metastasis	5	96	
45	56	F	Crypto Meningitis, Hydrocephalus	5	24	
46	38	F	Thoracic Meningioma	10	24	
47	57	F	Posterior Fossa Tumor	10	24	

## Results

Twenty-four men and 23 women, mean age 42.7 years (range: 20 to 67), were drained for a mean of 97.6 hours (range: 24 to 168) (Table 1). Diagnoses included skull base surgery (24), spontaneous and traumatic CSF fistulas (8), unintentional durotomy (7), intracranial hypertension due to meningitis (3), normal pressure hydrocephalus (2), pseudotumor cerebri (2), and surgery for a thoracic, intradural meningioma (1). The mean drainage rate was 8.3 ml/hour (range: 5 to 15). A single dose of prophylactic antibiotics was used in 35 patients. Due to surgeon preference, 12 patients received prophylactic antibiotics for 48 to 144 hours. 

Approximately 5 to 10 minutes were required to set up the system after placement of the lumbar drain. Thereafter, manual interventions to regulate flow were not required. Two patients (4%) reported headache during treatment. Drainage was stopped for eight hours and then restarted in one patient. The maximum hourly rate was decreased from 10 to 5 ml/hour in the other patient. Both reported resolution of headache. In one case, the prototype version of the device alarmed and required the replacement of a volume sensor. Accurate drainage was not interrupted, and there were no complications. There were no other mechanical or technical issues.

In 39 patients with iatrogenic or traumatic CSF leaks, the leak was successfully treated. In seven patients with suspected normal pressure hydrocephalus, post-infectious ventriculomegaly, or pseudotumor cerebri, we were able to assess the likelihood of improvement with shunting. The system, therefore, produced the “desired result” in 46 of our 47 patients (98%). One patient (2%) with undiagnosed hydrocephalus who had undergone a Chiari repair was drained for six days but, because of a persistent pseudomeningocele, required a reoperation to repair of the dural closure.    

## Discussion

Although lumbar drains are commonly used for traumatic and iatrogenic dural tears [3],^ ^after skull base surgery [2], and following some aortic aneurysm operations [11-12], they are risk-laden, require frequent nursing interventions, and are typically used only in an ICU setting. Houle, et al. developed an early, flow-regulated, electronic system using a device similar to an intravenous fluid pump [14]. It improved safety, eliminated over- and under-drainage, and required less manual oversight. Unfortunately, technical issues limited its adoption [2]. Attempting to address safety and cost issues, Nanidis, et al. recently published their experience with an inexpensive flow-regulated system [15]. The system was created from readily available components that were modified and assembled at the bedside. Although reportedly safer, assembly was difficult, and the system was not suitable for patients with labile intracranial pressure [15].

For patients requiring CSF drainage, most complications fall into one of three categories: over- and under-drainage, mechanical failure, and infection [13]. Over- or under-drainage can cause headache [4, 6, 13-14, 16-17], pneumocephalus [13-14], vision loss [13], brainstem herniation [3, 8, 16], intracranial hemorrhage [5, 18], and death [13, 16]. Mechanical failures related to the catheter included insertion issues, blockage and breakage [3, 7], numbness or nerve root pain [3-5, 13], and other insertion site problems, such as pain or bleeding [2]. Mechanical failures related to the tubing, collection system, and sensors included fractures or failures of the tubing or connectors, failures of the collection chamber, and failures of electronic equipment, including pressure transducers. Infectious complications included entry site infections [3, 5, 7] and meningitis [7, 9, 13].

Operator error or inattention most commonly caused over- or under drainage but could result in any of the above [19]. A summary of complications reported in recent review articles is presented in Table 2.

**Table 2:** Articles Included in the Review of Complication of External CSF Lumbar Drainage CSF - cerebrospinal fluid; NA - not applicable

		Açikbaş^[^^13]^	Ackerman^[^^16]^	Al-Tamimi^[^^7]^	Chotai^[^^4]^	Crowson^[^^17]^	Governal^[^^5]^	Grady^[^^20]^	Hoffman^[^^21]^	Houle^[^^14]^	Huang^[^^18]^	Klimo^[^^8]^	Marmarou^[^^6]^	Ransom^[^^2]^	Shapiro^[^^3]^	Sun^[^^9]^	Van Aken^[^^22]^	Totals	This Study
	Number of Patients	63	43	99	66	220	233	513	45	42	48	81	151	65	107	72	278	2,126	47
Over/Under Drainage	Headache or Nausea/vomit	7	13	NA		2	4	NA	NA	2	NA	NA	4		NA		NA	32	2
	Pneumo-cephalus	3								1								4	0
	Cranial Nerve Injury	1																1	0
	Herniation		1									3			3			7	0
	Hemorrhage/ Infarction						4				1							6	0
Mechanical	Catheter Failures	5				12	1					2	4	4	10			38	0
	Puncture Site Pain/Bleeding							1							1			2	0
	CSF Leak from Catheter Site			1				13						5				19	0
	Nerve Root Irritation	7			6		6	1							15			35	0
Infection	Local Wound Infection			1			2								3			6	0
	Meningitis	5		2		1	2	1	4			2	2		2	13	0	34	0
	Death	1	1															2	0
Efficacy	Number	59	41	77	59	200	34	NA	34	36	47	67	139	61	101	NA	NA	1025/1263	46
	Percent	94%	95%	78%	96%	90%	85%	NA	76%	86%	98%	80%	92%	94%	94%	NA	Na	81.2%	98%
Headache	Number	7	13	NA	0	2	4	NA	NA	2	NA	NA	4	0	NA	0	NA	32/955	2
	Percent	11%	30%	NA	0%	1%	2%	NA	NA	5%	NA	NA	3%	0%	NA	0%	NA	3.4%	4%
Compli-cations	Number	22	1	4	6	13	15	16	4	1	1	7	6	9	34	13	0	162/2204	0
	Percent	35%	2%	4%	9%	6%	6%	5%	9%	2%	2%	9%	4%	14%	32%	18%	0%	7.4%	0%

A list of possible articles was obtained using PubMed.com and the keywords “lumbar drain” and “complications”. Of 1,016 articles, 220 were listed as review articles. We next eliminated non-English language articles, those with incomplete data, and those for which a full-text copy was not available, leaving 125 papers. After eliminating non-neurosurgical articles, those dealing only with the treatment of increased intracranial pressure due to trauma, and those describing only the intraoperative use of a drain, 24 articles remained. We elected to summarize only those 16 articles with more than 40 patients. The number 40 was chosen so that reviewed papers would describe cohorts of similar size or larger than our subject population. The 16 identified studies, published between 1992 and 2015, described 2,126 patients and were believed to be representative. Of the 16, eight dealt with CSF leaks or potential leaks, five with possible normal pressure hydrocephalus patients, and in three, drainage was an adjunct for the treatment of subarachnoid hemorrhage. All listed complications were tabulated.

Complications were divided into over- or under-drainage (pneumocephalus, cranial neuropathies, herniation or near herniation events, and infarctions and hemorrhages), mechanical failures (catheter failures, wound site bleeding or drainage, spinal headaches after catheter removal, and spinal nerve root irritation), and infection. In cases of infection, we accepted the author’s categorization regarding false-positive cultures. Two deaths were reported. Not all authors tabulated complications identically and the reporting of headaches was especially variable. If a single patient suffered multiple complications, each complication was reported separately so the number of complications in this summary may exceed the number of patients with complications. Efficacy was as defined by the author and was not uniformly reported. Depending on the study, it usually implied the healing of a traumatic or iatrogenic CSF leak, the author’s belief that the lumbar drainage successfully identified those patients who would likely benefit from shunting, or the author’s assessment that lumbar drainage had resulted in lower intracranial pressures or made shunting unnecessary following aneurysmal subarachnoid hemorrhage. Acikbas, et al., for example, reported that lumbar drainage achieved what he called the “desired goal” when a CSF leak healed. In 94% of his patients, the leak healed but the complication rate, excluding headache, was 35%. Their mortality rate was 1.6% with their single death attributed to an infection [13]. Of our 47 patients, 39 with iatrogenic or traumatic CSF leaks were successfully treated and seven with suspected normal pressure hydrocephalus, post-infectious ventriculomegaly, or pseudotumor cerebri were successfully assessed prior to shunting (46 patients, 98%). This compares favorably with the outcomes described in the articles reviewed. 

Our 2% rate of headaches also compares favorably with results reported in the literature, and our rate of complications (other than headache) is lower. A prototype of the system required a sensor replacement, but patient treatment was not interrupted and there were no other mechanical issues. Patient comfort is rarely addressed in the neurosurgical literature but, in the authors’ opinion, is a frequently overlooked concern among patients. Since patient position would not affect drainage with FlowSafe, the head of the bed could be adjusted for comfort and restroom privileges were permitted. In our study population, the patients did not complain about activity restriction or comfort during drainage.

An ideal lumbar drainage system would be highly reliable, control drainage accurately, require little or no manual intervention, decrease the risk of complications, and decrease costs. Commercially available lumbar drainage systems do not require expensive equipment but rely on frequent nursing staff oversight and intervention. To manage flow, a staff member must open a stopcock at least hourly, remain at the bedside as CSF drains, and then close the stopcock manually when the desired volume is drained. Therefore, patients are generally managed in the intensive care unit (ICU) or step-down units. Our system does not require nursing interventions or an ICU level setting. Published estimates of daily ICU costs are approximately $4,335 per day compared to non-ICU daily costs of $2,132 [23-25]. In our institution, ICU beds cost approximately $4,000 more per day than ward beds. If our automatic system were used, stable patients requiring lumbar drainage would not require hourly interventions and could be treated in a non-ICU setting. As our mean duration of drainage was approximately four days, a potential savings of $8,800 to $16,000 per study patient could have been realized.

Some patients, such as those with suspected normal-pressure hydrocephalus (NPH), could potentially be drained at home. This would allow their caregivers to better observe and gauge the success of drainage improvement. The device used for most patients in this study was a prototype and only a small group of patients was involved. This study was designed for proof of concept. The current production version (Figure 1) is a small, integrated robotic controller consisting of a single module with a pressure transducer and more comprehensive alarm functions. Further studies are needed to better assess the FlowSafe system. We plan a larger trial with neurosurgery and spinal surgery patients. We also plan to study other patient populations, including neurosurgical patients requiring external ventricular drainage and in vascular surgery patients undergoing aortic repair who require lumbar drainage.

## Conclusions

In our experience, the FlowSafe system was reliable, convenient, safe, and effective. Our complication rate was lower than in published series describing currently available manual systems. Frequent nursing interventions were not required, and most patients requiring ELD could have been treated outside of the ICU. This would open beds for higher acuity patients and would result in significant cost savings. While this initial experience is encouraging, larger trials will be needed. Trials including vascular surgery patients treated with lumbar drainage during aortic repair surgery would also be appropriate.
